# Using Deep Learning for the Classification of Images Generated by Multifocal Visual Evoked Potential

**DOI:** 10.3389/fneur.2018.00638

**Published:** 2018-08-03

**Authors:** Nidan Qiao

**Affiliations:** ^1^Department of Neurosurgery, Huashan Hospital, Fudan University, Shanghai, China; ^2^Harvard Medical School, Boston, MA, United States

**Keywords:** mfVEP, artificial intelligence, neural networks, image classification, electrophysiology

## Abstract

Multifocal visual evoked potential (mfVEP) is used for assessing visual functions in patients with pituitary adenomas. Images generated by mfVEP facilitate evaluation of visual pathway integrity. However, lack of healthy controls, and high time consumption for analyzing data restrict the use of mfVEP in clinical settings; moreover, low signal-noise-ratio (SNR) in some images further increases the difficulty of analysis. I hypothesized that automated workflow with deep learning could facilitate analysis and correct classification of these images. A total of 9,120 images were used in this study. The automated workflow included clustering ideal and noisy images, denoising images using an autoencoder algorithm, and classifying normal and abnormal images using a convolutional neural network. The area under the receiver operating curve (AUC) of the initial algorithm (built on all the images) was 0.801 with an accuracy of 79.9%. The model built on denoised images had an AUC of 0.795 (95% CI: 0.773–0.817) and an accuracy of 78.6% (95% CI: 76.8–80.0%). The model built on ideal images had an AUC of 0.985 (95% CI: 0.976–0.994) and an accuracy of 94.6% (95% CI: 93.6–95.6%). The ensemble model achieved an AUC of 0.908 and an accuracy of 90.8% (sensitivity: 94.3%; specificity: 87.7%). The automated workflow for analyzing mfVEP plots achieved high AUC and accuracy, which suggests its possible clinical use.

## Introduction

Pituitary adenomas account for 15% of all intracranial neoplasms, making them one of the most common type of brain tumors (1). Patients report visual dysfunctions when tumors extend beyond the sella compressing the optic chiasm and nerve. Typical neuro-ophthalmic features include progressive loss of visual acuity and bilateral temporal visual field defect.

Visual evoked potential is used to evaluate patients with pituitary adenomas presenting visual symptoms. However, the efficacy of full-field visual evoked potential is limited by the fact that it provides a summed response of all the stimulated visual neurons. Recent development of multifocal stimulation techniques has resulted in the implementation of a new method for assessing visual functions, i.e., multifocal visual evoked potential (mfVEP), which is a unique approach for evaluating the visual pathway integrity. This technique dismisses the subjectivity of patients; thus, it is beginning to show promise in evaluating patients with compressive neuropathy (2). Several studies have shown that mfVEP can predict visual outcome in these patients (3, 4).

**Figure 1:**
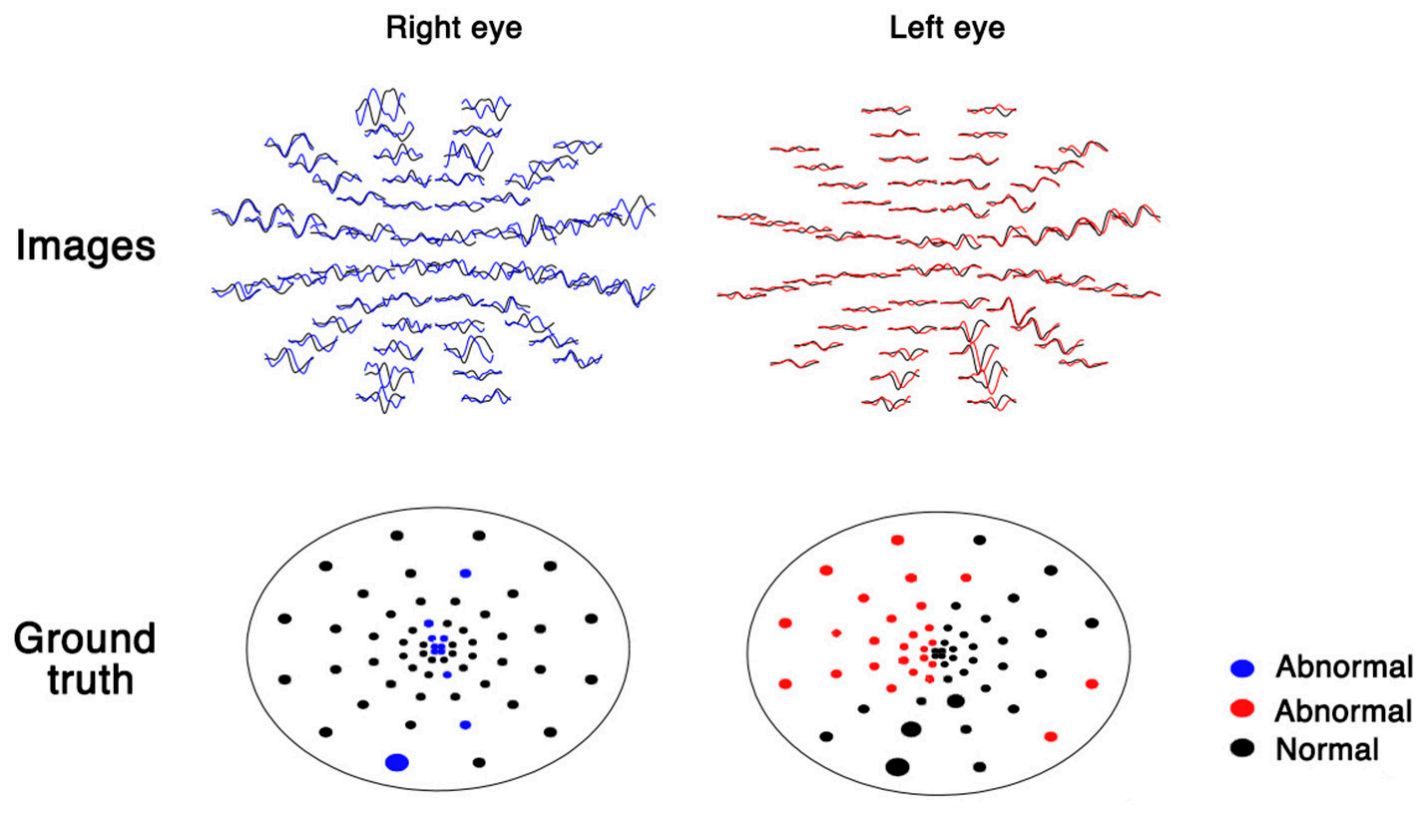
Multifocal visual evoked potential recordings in both eyes.

mfVEP generates 60 visual evoked potential images for both eyes (Figure 1). For the analysis of these images, a database of healthy volunteers is required. The analysis is time consuming (30 min by a trained ophthalmologist) and these images vary in qualities due to low signal-noise-ratio (SNR), which increases the difficulty during analysis. Recently, a few deep learning systems have shown high sensitivity and specificity for image classification (5, 6). Deep learning-based models also have shown great promise for denoising images (7). Moreover, from fundus to skin disease images, convolutional neural networks have shown to have great capability to distinguish images into multiple classifications (5, 6). Therefore, I hypothesize that deep learning-based automated workflow could facilitate the analysis and correct classification of these images by clustering ideal and noisy images, denoising images using an autoencoder algorithm, and classifying normal and abnormal images using a convolutional neural network.

## Methods

All the procedures followed the tenets of the Declaration of Helsinki, and the study was approved by Huashan Hospital Institutional Review Board; written informed consents were obtained from all participants.

mfVEP recordings were obtained as in my previous studies (2). The stimulus was a 60-sector, cortically scaled dartboard pattern with a mean luminance of 66 cd/m^2^ and a Michelson contrast of 95%. The dartboard pattern had a reversal frame rate of 75 Hz. The patients were instructed to fixate on the center of the dartboard pattern (marked with an “X”) with their best refractive correction; the eye position was monitored continuously. Three recording channels were connected to gold cup electrodes. For the midline channel, electrodes were placed 4 cm above the inion (active), at the inion (reference), and on the earlobe (ground). For the other two active channels, the same ground and reference electrodes were used, but the active electrode was placed 1 cm above and 4 cm lateral to the inion on either side. Two seven-minute recordings from each eye were obtained and the averaged responses were used. Normal or abnormal rating was given by comparing each sector with the same sector for healthy volunteers using MATLAB programs (MathWorks, Natick, MA, USA).

As part of the automated workflow, images and ground truth were extracted from the mfVEP report using a Python-based algorithm. The plot of each sector was transformed into an image of 30 × 50 pixels, which yielded 120 images for each patient (60 images per eye, as shown in Figure 1).

A convolutional neural network was used to classify the images into normal or abnormal images. The convolutional neural network algorithm computes the likelihood of abnormality from the intensities of pixels in each image. Training this algorithm requires a large set of images in which the ground truth is already known (training set). During the training process, the parameters of the neural network are initially set to random values. Then, in each training step, the algorithm compares the calculated likelihood with the ground truth and modifies the parameters slightly to decrease the error. The algorithm repeats this process for every image in the training set for several iterations. Finally, the algorithm learns how to compute the correct likelihood from the pixels of all images in the training set with the least error. A convolutional neural network (VGG19 architecture, Supplement Table 1), which learns to recognize the amplitude or latency of mfVEP using local features, was used in this study.

For denoising noisy images, an autoencoder algorithm was used. An autoencoder is a neural network that is trained to attempt to match its input to its output. The network may be viewed as consisting of two parts: an encoder block that encodes the images and a decoder block that performs reconstruction. Two convolutional layers that were symmetrically arranged in both encoder and decoder blocks were used for the denoising process (Supplement Table 2). The training process is same as that of the convolutional neural network algorithm described earlier. First, noisy images and ideal images are used to train the autoencoder algorithm. Then, denoised images are obtained by the trained autoencoder algorithm with noisy images as inputs. Finally, another convolutional neural network is trained for classification of the denoised images.

To speed up the training process, pre-initialization weights from the same network trained to classify objects in the ImageNet dataset were used; random dropout was used to prevent overfitting. The dataset was divided randomly into three parts: (1) training: 80% of the data was used to build the algorithm; (2) validating: 10% of the data was used to optimize the hyperparameters; and (3) testing: 10% of the data was used to test the algorithm for an unseen dataset. The performance of the algorithm was measured using the area under the receiver operating curve (AUC) by plotting sensitivity versus 1-specificity in the testing set. The final performance was achieved by 10-fold cross validation (the previous process was repeated 10 times). All the analyses were performed on Python 3.6 with the Keras package.

## Results

**Figure 2:**
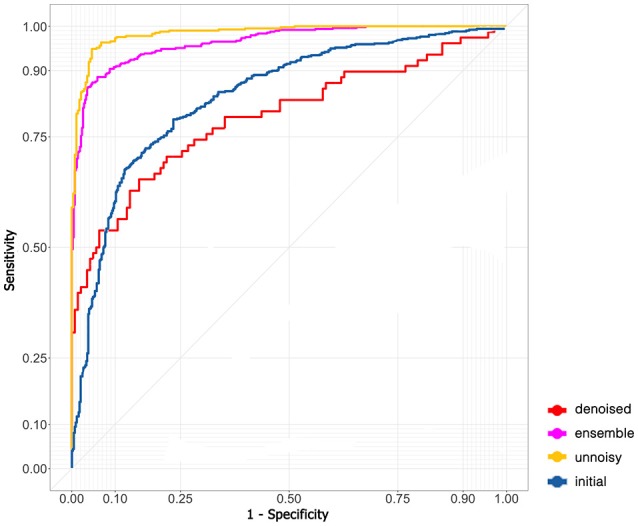
ROC curve of different models.

A total of 76 mfVEP examinations were included in this study. All the participants had suprasellar tumor. Among the 152 eyes examined, mfVEP was abnormal for 140 eyes. In the initial algorithm, 9,120 images (4,912 normal images and 4,208 abnormal images) were used. The training dataset contained 7,296 images; both validation and testing datasets contained 912 images. The AUC of the initial algorithm in the testing set was 0.801 (Figure 2) with an accuracy of 79.9%. The sensitivity was 73.9% and the specificity was 85.1%.

The model did not perform very well even with the state-of-art deep learning architecture. I assumed that the problem was due to the existence of many noisy images and therefore excluded noisy images with low SNR and fitted the remaining images to the model again (5,530 images in the training dataset and 691 images in both the validation and testing dataset). The performance in the testing set skyrocketed to an AUC of 0.985 (95% CI: 0.976–0.994, Figure 2) and an accuracy of 94.6% (95% CI: 93.6–95.6%). The sensitivity was 94.8% (95% CI: 93.2–96.4%) and the specificity was 94.9% (95% CI: 92.9–96.9%).

An autoencoder algorithm to denoise noisy images (2,208 images) was used. The denoised images (1,768 images in the training dataset and 220 images in both the validation and testing dataset) were fitted to the convolutional neural network. The acquired AUC in the testing dataset was 0.795 (95% CI: 0.773–0.817, Figure 2) and the accuracy was 78.6% (95% CI: 76.8–80.0%). The sensitivity was 94.4% (95% CI: 92.4–96.4%) and the specificity was 50.0% (95% CI: 45.5–54.5%).

The ensemble model combined the previous results of ideal images and denoised images to obtain the final AUC (0.908, Figure 2) and accuracy (90.8%). The sensitivity and specificity were 94.3 and 87.7%, respectively.

## Discussion

I built an automated workflow for analyzing the images of mfVEP. The workflow can extract images from each sector, convert noisy images to denoised images, and predict abnormality in the images. The workflow can reduce the analysis time to the greatest extent. Improved accuracy was observed after separating noisy data from ideal data. Ten-fold cross-validation results of the model suggest that the model is robust.

By combining models built on ideal images and those built on denoised images created by the autoencoder algorithm, high AUC and accuracy were achieved, suggesting possible clinical use. Moreover, saved parameters and structures of the trained neural network can be used in other institutions without healthy controls.

However, this study had some limitations. The diagnostic test being studied, mfVEP, is not widely available and is time consuming. The lack of other clinical data such as visual field or optical coherence tomography may also limit the possible clinical utility, although previous researches have published these data (2, 8). Unfortunately, ground truth is needed for the metrics to determine the quality of the model. Some ground truth values may be incorrect due to noise. Unsupervised learning and model development should be applied and evaluated in future studies. Moreover, only patients with compressive optic neuropathy were included in this study. Further research is necessary to evaluate the applicability of the deep learning system for other ophthalmological diseases and the utility of the deep learning system to improve vision outcomes.

## Author contributions

The author confirms being the sole contributor of this work and approved it for publication.

### Conflict of interest statement

The author declares that the research was conducted in the absence of any commercial or financial relationships that could be construed as a potential conflict of interest.
