# Supplementary material for: Using Deep Learning for the Classification of Images Generated by Multifocal Visual Evoked Potential
Source: Front Neurol. 2018 Aug 3;9:638. doi: 10.3389/fneur.2018.00638 (PMC6085441; doi:10.3389/fneur.2018.00638)
Supplement: Supplementary file 1 [file Table_1.DOCX]

Supplement Table 1. Structure of VGG19

_________________________________________________________________

Layer (type) Output Shape Param #

=================================================================

input_5 (InputLayer) (None, 50, 50, 3) 0

_________________________________________________________________

block1_conv1 (Conv2D) (None, 50, 50, 64) 1792

_________________________________________________________________

block1_conv2 (Conv2D) (None, 50, 50, 64) 36928

_________________________________________________________________

block1_pool (MaxPooling2D) (None, 25, 25, 64) 0

_________________________________________________________________

block2_conv1 (Conv2D) (None, 25, 25, 128) 73856

_________________________________________________________________

block2_conv2 (Conv2D) (None, 25, 25, 128) 147584

_________________________________________________________________

block2_pool (MaxPooling2D) (None, 12, 12, 128) 0

_________________________________________________________________

block3_conv1 (Conv2D) (None, 12, 12, 256) 295168

_________________________________________________________________

block3_conv2 (Conv2D) (None, 12, 12, 256) 590080

_________________________________________________________________

block3_conv3 (Conv2D) (None, 12, 12, 256) 590080

_________________________________________________________________

block3_conv4 (Conv2D) (None, 12, 12, 256) 590080

_________________________________________________________________

block3_pool (MaxPooling2D) (None, 6, 6, 256) 0

_________________________________________________________________

block4_conv1 (Conv2D) (None, 6, 6, 512) 1180160

_________________________________________________________________

block4_conv2 (Conv2D) (None, 6, 6, 512) 2359808

_________________________________________________________________

block4_conv3 (Conv2D) (None, 6, 6, 512) 2359808

_________________________________________________________________

block4_conv4 (Conv2D) (None, 6, 6, 512) 2359808

_________________________________________________________________

block4_pool (MaxPooling2D) (None, 3, 3, 512) 0

_________________________________________________________________

block5_conv1 (Conv2D) (None, 3, 3, 512) 2359808

_________________________________________________________________

block5_conv2 (Conv2D) (None, 3, 3, 512) 2359808

_________________________________________________________________

block5_conv3 (Conv2D) (None, 3, 3, 512) 2359808

_________________________________________________________________

block5_conv4 (Conv2D) (None, 3, 3, 512) 2359808

_________________________________________________________________

block5_pool (MaxPooling2D) (None, 1, 1, 512) 0

=================================================================

Total params: 20,024,384

Trainable params: 20,024,384

Non-trainable params: 0

_________________________________________________________________

Supplement Table 2. Structure of autoencoder algorithm

_________________________________________________________________

Layer (type) Output Shape Param #

=================================================================

input_1 (InputLayer) (None, 30, 50, 3) 0

_________________________________________________________________

conv2d_1 (Conv2D) (None, 30, 50, 16) 448

_________________________________________________________________

max_pooling2d_1 (MaxPooling2 (None, 15, 25, 16) 0

_________________________________________________________________

conv2d_2 (Conv2D) (None, 15, 25, 8) 1160

_________________________________________________________________

up_sampling2d_1 (UpSampling2 (None, 30, 50, 8) 0

_________________________________________________________________

conv2d_3 (Conv2D) (None, 30, 50, 3) 219

=================================================================

Total params: 1,827

Trainable params: 1,827

Non-trainable params: 0

_________________________________________________________________
